# The Increase of Insanity and Some of Its Causes*Read before the Indiana State Medical Society, June 6, 1895.

**Published:** 1895-09

**Authors:** S. E. Smith

**Affiliations:** Richmond, Ind., Medical Superintendent of the Eastern Hospital for the Insane


### THE INCREASE OF INSANITY AND SOME OF ITS CAUSES.*

BY S. E. SMITH, M. S., M. D., RICHMOND, IND.,
Medical Superintendent of the Eastern Hospital for the Insane.

I do not believe in the decadence of the human race. A broad view of man and his environment induces in my mind no misgiving or fear of his future. It is not to be denied, however, that in the progress of civilization, which is nothing more nor less than mental evolution, errors of growth creep in and centers of decay appear. Such may originate in faulty dogmas and systems of education, neglect of hygieneic laws, and the vicious customs and unfortunate accidents of life. Be their origin what it may, these malformations and tendencies exist and are evils. We must recognize them as such. From small beginnings they thrive and engraft themselves upon the social body in the form of dependents and barnacles, which, by multiplication, become a burden and hindrance to growth and prosperity. To eliminate them wholly and have left a complete human family with perfect members, is an ideal state, to which we may not hope to attain.

While eradication is impossible, we can do something towards the prevention of extensive contamination and, by the discovery and application of corrective influences, nullify, to some extent at least, the malignant character of these evils, not the least of which is insanity.

The etiology of insanity presents a perplexing problem, but it is a subject of the gravest import, and affecting the welfare of society as deeply as it does, is worthy of the most thoughtful consideration. To the medical profession the public looks for its solution.

The general impression to the effect that diseases of the mind are increasing rapidly, is erroneous. A marked increase is apparent, I grant, but it is in the main relative, and only to a light extent actual.

The whole country considered, there was in 1880, one insane to 545, and in 1890 one to 589—a relative decrease. The new

*Read before the Indiana State Medical Society, June 6, 1895.

states of the northwest, on account of the large number of foreigners, exhibit large additions to the insane classes. The Scandinavians, particularly, are, in America, prone to insanity. In the State of Minnesota, they form 16 per centum of the population, and yet furnish 28 per centum of the insane.

The lunacy commissioners of England and Scotland claim only an apparent increase in these countries. In Ireland, an actual increase is undoubted, and there is one insane to 289 of population. While the facts are serious enough, they do not conform to the alarming popular view.

In his attempts to discover the many pathogenic factors of insanity, the American alienist is beset by many difficult obstacles. A conglomerate population, representing every tongue and land, and every rank and grade of the social world, confronts him. Many of his subjects were conceived, reared and trained, if trained at all, under the influences of peculiar institutions and unknown surroundings. These factors multiply the lines of his investigation without at the same time increasing his facilities for inquiry. Family histories, so essential to his researches, cover, usually, one, sometimes two and rarely three generations before he is lost beyond the seas. In many classes the stigma attaching to insanity, which only the broadening influences of education can remove, encourages relatives and friends to conceal from the inquirer facts and data of prime importance. A yet greater barrier to the student of physical phenomena, hindering his step, although it may promise the final one, is the state of opinion relative to *post-mortem* examinations. The future promises to relieve us, at least to some extent, of this hindrance.

Having granted a slight increase of insanity, one of two conditions, if not both, certainly exists. Either our brain of to-day is less stable than that of our forefathers, or the stress of environmental forces is more severe. That the present brain is more delicate and more unstable is not improbable or incomprehensible. It follows in the line of the dictum that evolution progresses "from the homogeneous to the heterogeneous, from the stable to the unstable." The evidence that the brain is the result of a long process of development is unmistakable. The highest form of organization is psychical phenomenon. Ascending through the cord and brain, each step becomes more intricate and the functions correspondingly more complex until the cortex is reached. Here in the cortex, with its infinite number of cells, and countless intercommunicating fibers, we find the most sensitive and complex arrangement of organized matter, the highest form of specialization. Here at the cortex, the region of greatest complexity, we find the function of the highest potentiality—the function of thought.

The same may be said of the entire organism of man. As he advances, as his ability to overcome external forces and to subvert them to his own use enlarges, he becomes more complex and delicate anatomically. At the same time his powers of resistance to forces, elements, conditions, which formerly affected him slightly, if at all, have diminished. The great American trotter and Jersey cow, representing remarkable degrees of development in certain directions, are less rugged and more susceptible of disease than their antecedents. They are more complex, more capable and more sensitive. It is likewise true of all organic life. Evolution is the process of increasing complexity and capability, and no better example of this procedure is to be found than in the nervous system.

Accepting the proposition that man is growing more complex and sensitive, the ultimate step of the evolving process would appear to be complete annihilation. But here, as always, in every form and step of life, for every hardship, every obstacle and every change in condition there is a compensatory influence which makes continued existence certain, easier and better. The law of compensation is ever operative. Now the compensatory influence for the increasing complexity of the human brain, with its greater liability to maladjustment, is a mollification of the stress of environment. So long as these two conditions are complementary all is well and our advancement in the direction of greater mentality is assured and positive, and abnormalities do not arise. Unfortunately, however, this state of equilibrium does not exist. It is an ideal state; approachable, but not attainable. The general tendency is towards it, but here come periods when proximity is greater or less. To-day our state of civilization has them markedly out of balance. It matters not on which side of the scales we choose to locate the error. Call it as we may, an excess or a deficit, the fact remains—the stress of environment is too severe for our potential, yet delicate and susceptible cortex.

That all psychical phenomena are referable to the cortex, we must admit. Whatever our personal views may be of the mind and its action, whether we accept it as an entity co-existent with, yet independent of, cortical, cellular and molecular change, or whether on the one hand cause, or on the other effect, of such change, we must defer to the position that there is no mentality without cortical alteration. It follows then that for every psychical phenomenon there is a nervous phenomenon, and for every psychosis there is a physical basis. All the bases of insanity in its many forms are not demonstrable. A few only are. Some forms of cellular changes are evident, but molecular alterations are as yet beyond our reach.

We call those varieties of insanity in which pathological lesions are appreciable, organic, and those dependent upon molecular derangement or delicate cellular disease, incapable of demonstration, functional. According to our hypothesis, that all the psychoses have some physical basis, even the so-called functional diseases may be considered structural. I feel that the psychiatrist of the future will turn his studies in the direction of molecular physics and am confident enlightenment will follow along this line.

Given a highly specialized, an unstable brain, as I have tried to demonstrate, under a severe stress of surrounding forces, the pathogenesis of insanity becomes in great part clear. It is a vulnerable organ, susceptible to influences and diseases which the inferior and simple brain was able to resist. Modern education and training have been almost altogether mental. Little or no attention has been given to physical protection against morbific and adverse forces during the process. This is one of the errors of a splendid system of education which is now being corrected.

We are living in a very active age and at a rapid rate. It is a period of vaulting ambition. Every man is straining every effort to outdo his neighbor. The man with an income of one thousand dollars wants one of five, and he of five wants ten, and so on *ad infinitum*. It is a rushing, mad struggle to reach the top of the ladder. The time was when the old dictum "There is always room at the top" was truth, but to-day it is not applicable. It is now even crowded at the top, and human effort is, I fear, directed toward raising the ladder. Contentment is no longer a part of us. We have forgotten that contentment means happiness and health. We violate every hygienic law known to science and boast of our ability to do it. We sit at our desks in our offices, business houses or factories for ten, twelve or fourteen hours a day, eat cold lunches on the run to save a dollar's worth of time, discharge our social obligations during the first half of the night and even take our business to bed with us in order to shorten the five or six hours left for rest, although tired nature is clamoring for ten. All this effort indicates an enormous expenditure of nervous energy. The reserve nerve force is not husbanded. The demand continues, a last struggle is made, collapse follows. This is the stress of environment—too severe for an unstable brain. These two conditions account for many cases and many types of insanity and kindred affections. Some of its products of a mild character are covered by the very familiar and popular expression "Nervous prostration," and the more fashionable disease, "Neurasthenia," is another offspring.

The insane diathesis is either inherited or acquired. To the transmission from parent to offspring of nervous defects favorable to the development and manifestations of the psychoses, we are indebted for the greatest etiological factor. Not all children of insane parents become insane, but many do. Subjects of the inherited insane diathesis, as unmistakable as the color of the skin, may pass a quiet, uneventful life, eccentric though it be, without the shock of an exciting cause and escape. Again, we observe every child of the family is affected. Recently I had under my immediate care, out of four offsprings of the same parents, two sisters and a brother, and was compelled to stop the visits of the third daughter to the affected three on account of her disposition to mental disturbance. Another instance in the Eastern Hospital is a brother, sister and two cousins. It is observable that the daughters of an insane mother are more apt to manifest the disease than the sons, and that the father is more liable to transmit his nervous defects to his sons. I have now in my charge both a father and his son manifesting very similar forms of mental disease. The mother and two daughters are, to all appearances, unusually vigorous, both in mind and body. In another instance, are two daughters. Recently I dismissed as recovered a woman, a typical case of melancholia with delusions of fear, and in less than a month received her only daughter as a typical case of puerperal mania. Her infant, and female at that, will probably have a like experience. In another instance, while a father and son were in my care, three other sons committed suicide and the fifth became a murderer. There were no daughters, to my knowledge in this family. All hospitals for the insane furnish similar records.

I am unable to subscribe to the generally accepted law of heredity, that mental alienation, as well as other transmitted abnormal tendencies, appear at approximately the same age in the several generations. My observations are to the effect that manifestations of mental deterioration are disposed to occur at an earlier period in each succeeding generation. A parent epileptic, if the disease is of non-traumatic origin, is more liable to convey to children a neurotic tendency than any form of the neurosis. If conception has occured after convulsions have appeared, we may, with a reasonable degree of certainty, depend upon its reappearance. Even in traumatic epilepsy, as in acquired diathesis, we occasionally see transmission of a constitutional taint. That the dangers of hereditary contamination are vastly augmented goes without saying, when both parents are insane. Cases of what the French term *folie a deux* are rare, it is true, but instances of insanity in one parent and an unmistakable insane diathesis in the other, are more frequent. The progeny of such a pair has no escape. The imperfect products of consanguinity belong to the insane diathesis and the number is no small aggregate. Likewise, marriages of expediency favor the development of constitutional nervous defects. Especially in the populous centers, such unions, under artificial conditions of life, are common and increasing. Nature's plan of bringing compatible temperaments together has to be laid aside, and convenience substituted. A similar evil follows the modern tendency to shift the responsibility of the propagation of the species by the best fitted to the unfit. When the period comes, if ever, when we will propagate our kind with some wisdom and discretion, as we now do our horses and dairy herds, there will be fewer defectives.

I regret my inability to state with a reasonable degree of exactitude the number of insane in which hereditary diathesis is observable. The statistics of public and private hospitals, to which we must look for the figures, are not sufficiently uniform for the purpose. It is certainly safe to say that fifty per centum of the cases coming under the observation of the alienist belong to this class. These are cases that furnish the large number of chronic insane that fill our lunatic asylums, and are ever dependent upon society.

The other cases belonging to the class of the acquired insane diathesis, are less in number, are amenable to treatment, and under favorable conditions, furnish the twenty, twenty-five and even the thirty percentage of recoveries we are able to report.

At this point I desire to make a distinction between idiocy and imbecility, and insanity. The fact that in some of our states both classes are admitted into the State hospitals for insane, disposes to error and confusion in the matter of statistics, and consequently, in deductions. Idiocy and imbecility are, undoubtedly, closely allied to the inherited insane diathesis, and the line of demarcation between them is probably no more clearly defined than that between insanity and mental health; yet they form two distinct and separate classes to be borne constantly in mind. Idiocy and imbecility of the one class represent states of arrested development, and the other, diseases of the cortex. We may reasonably say, that when a brain comes into this world, there is innate in it a given potentiality to develop, a certain degree of maturity to reach, a fixed amount of energy to evolve. If for any reason this process of evolution is stopped permanently, we have, depending upon the stage of arrest, idiocy or imbecility. On the other hand, insanity is a disease of the cortex, and it matters not whether that disease seizes upon a mature or immature brain. Hence, an imbecile may become insane.

There is one other predisposing cause. It might safely be placed under one or the other heretofore mentioned, but on account of its gravity, I prefer to state it separately. It is, the nullification of the law of natural selection. Upon the principle that in union there is strength, men through all time have banded themselves together for mutual benefit and protection. In these groups the strong protect the weak. Two are enabled to live where one too deficient to stand alone would otherwise be lost in the struggle for existence. These weaklings not only live but procreate their kind. They are the beneficiaries of society. They are abnormal both in body and mind and contribute largely to our insane population. The dependent classes are thus magnified. It is here that the operation of the law of the survival of the fittest is suspended. Charity and humanity, the loveliest attributes of the highest development, have come into power and repealed that law, substituting for it the "law of reciprocity, the loftiest ethic of which mankind is capable." I do not underestimate the value and beauty of the beneficence of our charity organizations, when I say, they contribute so largely to the conservation and multiplication of these imperfect beings. God forbid that I should utter a single word in disparagement of these benevolent groups which exist in obedience to the divine injunction, "Love thy neighbor as thyself," but this result we cannot fail to recognize.

Neither is it necessary for me to apologize for saying that insanity is a product of education. It seems harsh, but the fact remains and must be evident to every broad observer. I must not be understood, however, to declare that the classes of the highest education and development furnish the most insanity. On the contrary, they furnish even less proportionately than others. The masses are the chief source of supply. Our observation that insanity is a product of civilization still holds good, because education is not confined to the advance guard. All classes are progressing—some rapidly, others slowly, yet all are advancing. Development of the leaders implies elevation of the masses, with its resultant unstability of the brain. Individual education is the ideal plan of mental and physical training. Being impracticable, mass education is our only alternative. Among the most serious evils is the absolute impossibility of the establishment of a standard amount of mental exercise, just to every one of a given number of pupils. Too often the leaders, the brightest and most active, encouraged by promises of promotion and honors, determine it, to the detriment of the majority, who suffer from too rapid advancement. The system of "cramming" is hurtful and vicious. Every schoolboy and college man has felt the strain and exhausting mental effort of the quarterly examinations. These methods produce a degree of mental tension harmful to well-rounded and healthful psychic development. Likewise is specialization in the early process of education deleterious. The expansion of particular faculties of the mind in tender years begets a symmetry in the adult. Musical and mathematical prodigies do not result from broad and generous mental discipline. Precocity requires a judicious inhibition. What we want, and what we need, is a training of muscles as well as mind, symmetrical in all its details; and we will get it. I have faith in the future.

We have mentioned the three great predisposing causes of insanity. All others are incidental, and may be regarded as exciting only. Intemperance, diseases such as syphilis, tuberculosis, typhoid and the like, traumatism, shock, gross brain disease and what not, may be included in this class. That alcoholism is a potent factor in the excitation of insanity is undeniable, but that it produces the large proportion of the cases which is popularly claimed, I am unable to affirm. Alcoholism is more prevalent in the large centers of population, and it is there that its effects upon the human organism are best studied. In the agricultural districts of our western states, it cannot be looked upon as one of the great causes of insanity. In a recent special report of the inspectors of lunacy in Ireland, twenty out of twenty-two medical superintendents of district asylums agree that the proportion of cases of lunacy due to alcohol varies from 10 to 35 per centum of the total number of admissions. Such a high rate is not found in America, and probably in no land save Ireland.

Eight per centum is a liberal estimate of the cases in the Indiana hospitals. Alcohol seems to have a special deleterious effect upon the Irish people. Dr. Dana, of New York, observes in "Alcoholism and the Classification of Inebriates," 1893, that out of 315 cases admitted in the special department of Bellevue hospital, 180 or 56 per centum were Irish and of Irish descent, and also that the preponderance of Irish has always been marked, but grows gradually less as the Irish become Americanized.

As to syphilis, I believe it more potent as a cause of mental and nervous disease than we have ordinarily estimated. In my observation of several hundred cases of lunacy, about 12 per centum have shown a history of syphilis. In general paresis and the various forms of the scleroses is a history of syphilitic infection more frequent. In a note on the subject of general paresis made recently, I stated that 25 per centum of the deaths, prior to the date of the statement, in the Eastern Hospital were due to general paresis and its complications, and in the search for the cause or combination of causes of this disease the fact that 40 per centum of the cases show a history of syphilis, must not be overlooked. The argument so often advanced, that syphilitic contamination is not an essential cause of insanity, because the Alaskans, native Hawaiians and American Indians are being annihilated, and the negro race, seriously affected by it without concomitant mental diseases, is not to my mind sound. The facts harmonize with the hypothesis that the highly specialized brain is less stable and more susceptible to morbid invasion than the barbaric brain. Consequently, syphilis and kindred morbific factors may exist, however largely, in savagery with its narrow mental field, without the manifestation of complex nervous symptoms. As to the negro race, the psychoses in it are rapidly increasing. In 1860, in the State of Georgia, there were forty-four insane negroes, one insane negro to every 10,584 of the population, and in 1880, there was one to every 1,764. Twenty years ago there was not a record of a single case of general paresis in the colored male, and five years ago not one reported case in a colored female. Within the past year, fifteen colored men in one institution in the South are reported to have general paresis, and I recall five cases during the same period to have been reported in colored women.

Ten years ago as I came fresh from the schools, I had faith in the curability of syphilis. I am not ready now to deny its curability, but am frank to confess my faith grows less as I see more of the disease of the brain. By accident, I have lately come in contact with a few hopeless lunatics suffering from general paresis and gross brain disease, who, six years before, were discharged as cured of syphilis after twelve months' treatment. While such observations are not sufficient on which to base a scientific conclusion, they test a faith less than Abrahamic in character.

Immigrants are drawn mainly from the peasant classes of Europe. They come to America deluded by false promises, and sanguine of acquiring wealth and leading lives of luxurious ease. Disappointed, and without sympathy and assistance of friends and relatives, and without sufficient means to return them to the land of their birth, they fall into a condition of mental depression. Many of them, no doubt, are already victims of inherited neurotic tendency, and the shock from the circumstances in which they find themselves is not infrequently sufficient to develop insanity. The change in the character of their diet and customs has, also, its influence. The major portion of them were formerly accustomed to the coarser vegetables, and little in the way of albuminoids. Turnips, cabbage, and the like, are in Northern Europe the chief articles of subsistence. The coarse black-bread found in the peasant's hut of this section is not readily forgotten by an American who has partaken of it. Here they subsist largely upon meats, and doubtless are given more to excess in both eating and drinking. There are, also, reasons for the belief that among the vicious class which foreign countries, for selfish reasons, transport to our country, there are many insane. I remember to have seen one Swede admitted to one of our hospitals still wearing the clothing, bearing the mark of a Swedish asylum. Others coming within my observation were, although recent arrivals, suffering from chronic mental disease of many years standing. From the number of Poles and Danes found in the northern institutions one is led to believe there is more than one "Melancholy Dane," through whose "Ivory Gate"

"Pluto sent delusive dreams."

Another exciting cause of some importance is a class of unwholesome literature which is distributed broadcast throughout the land. There are daily papers and periodicals, and I am glad to say the number is not large, that reek with sensationalism and crime, which are mischievous, in that they encourage evil and wrongdoing by suggestion. Likewise, many books, some by authors of more or less repute, may be objected to for the reason that they deal too much with morbid conditions. As long as readers of such are confined to those of mature and cultivated minds, who are able to enjoy and profit by a volume on account of the author's purity of diction or elegance of style, independently of his vicious principles, little harm can come of them. But, unfortunately, the youth and the masses are incapable of reading in this manner. The thought expressed is the feature which most impresses the ordinary reader. However, the serious effects of such literature are too well appreciated to require any discussion here, and what ever bearing it may have upon nervous temperaments, it is indirect, and, through these inculcations of doctrines, inimical to the highest standard of social excellence. The publications to which special reference is made are those of a quasi medical character, issued by charlatans, who are found by the score in all our large cities. These imposters allure their victims by attractive advertisements and delude them by fairy stories of their wonderful skill, medical and surgical, and offer as proof innumerable testimonials of persons without existence. Their chief prey is the neurotic and hypochondriacal, whom they rarely, if ever, see, preferring to communicate with them through the mails by letter, circular and printed matter. This constitutes the literature, if it may be dignified by such a name, that is so baneful. The best of it is horrible, and the impression it makes upon the minds of credulous and feeble-minded individuals is almost beyond belief. By graphic description it incites in these people a fear of impending insanity and physical decay. It names and magnifies as the warning notes of the imminent crisis the signs and symptoms of slight ailment; and the confiding patient, by constant study of self, feels himself, by every ache and pain to which all human flesh is heir, borne one step nearer the dreadful abyss. Progress from this awful illusion to hallucination and delusion is rapid. I do not claim that such literature produces insanity *de novo* but I do claim it gives impetus to native tendency which, under favoring conditions could have been avoided. The cases are many, and every reputable physician numbers in his clientele several patients thus victimized, whom they relieve, if at all, only with greatest difficulty.

